# Dexamethasone Loaded Liposomes by Thin-Film Hydration and Microfluidic Procedures: Formulation Challenges

**DOI:** 10.3390/ijms21051611

**Published:** 2020-02-26

**Authors:** MD Al-Amin, Federica Bellato, Francesca Mastrotto, Mariangela Garofalo, Alessio Malfanti, Stefano Salmaso, Paolo Caliceti

**Affiliations:** Department of Pharmaceutical and Pharmacological Sciences, University of Padova, Via F. Marzolo 5, 35131 Padova, Italy; md.alamin@studenti.unipd.it (M.A.-A.); federica.bellato@phd.unipd.it (F.B.); francesca.mastrotto@unipd.it (F.M.); marinagela.garofalo@unipd.it (M.G.); alessio.malfanti89@gmail.com (A.M.); paolo.caliceti@unipd.it (P.C.)

**Keywords:** liposome formulation, microfluidic technique, dexamethasone loaded liposomes, controlled release

## Abstract

Liposomes have been one of the most exploited drug delivery systems in recent decades. However, their large-scale production with low batch-to-batch differences is a challenge for industry, which ultimately delays the clinical translation of new products. We have investigated the effects of formulation parameters on the colloidal and biopharmaceutical properties of liposomes generated with a thin-film hydration approach and microfluidic procedure. Dexamethasone hemisuccinate was remotely loaded into liposomes using a calcium acetate gradient. The liposomes produced by microfluidic techniques showed a unilamellar structure, while the liposomes produced by thin-film hydration were multilamellar. Under the same remote loading conditions, a higher loading capacity and efficiency were observed for the liposomes obtained by microfluidics, with low batch-to-batch differences. Both formulations released the drug for almost one month with the liposomes prepared by microfluidics showing a slightly higher drug release in the first two days. This behavior was ascribed to the different structure of the two liposome formulations. In vitro studies showed that both formulations are non-toxic, associate to human Adult Retinal Pigment Epithelial cell line-19 (ARPE-19) cells, and efficiently reduce inflammation, with the liposomes obtained by the microfluidic technique slightly outperforming. The results demonstrated that the microfluidic technique offers advantages to generate liposomal formulations for drug-controlled release with an enhanced biopharmaceutical profile and with scalability.

## 1. Introduction

Almost 60 years ago, Alec Bangham described liposomes as swollen phospholipid systems [1,2]. The era of drug delivery using lipidic vesicles had begun. Liposomes represent a versatile drug delivery system that allows for the encapsulation of a variety of active compounds either in their lipid bilayer or in the aqueous core [3]. Doxil^®^ developed by Barenholz and Gabizon [4] for the treatment of Acquired Immune Deficiency Syndrome (AIDS) related Kaposi sarcoma has been the first liposomal formulation approved by the Food and Drug Administration (FDA). Despite extensive research in this area, to date only 15 liposomal formulations are in clinical use [5], along with the first generic version of liposomal doxorubicin hydrochloride (Lipodox^®^). The approval of this generic formulation by the FDA was promoted by the shortage of Doxil^®^ in late 2011 due to manufacturing and regulatory hurdles, which speeded up the approval of Lipodox^®^ produced by Sun Pharma. On the other hand, the Doxil^®^ shortage raised also awareness about the issues of pharmaceutical manufacturing of liposomes and the need of novel procedures for large scale production of products with a high standard of pharmaceutical quality. Indeed, the manufacturing of liposomes presents certain major issues to be addressed: i) batch to batch variability, ii) control over the size of the liposomes, iii) the number of steps required for drug loading, iv) time-consumption and the complexity of production and v) the scalability of the process.

Over the past few decades, different approaches have been proposed to manufacture liposomes. These include lipid film hydration, reverse phase evaporation, ethanol injection, ether injection, detergent removal, etc. [6,7,8,9,10]. Most of the methods used to formulate liposomes require additional post processing steps to increase the liposome homogeneity in term of lamellarity and size, such as freeze-and-thaw cycles, extrusion, and sonication [11,12]. Liposome size and size distribution are key features for in vivo applications, being among the main parameters dictating the biopharmaceutical behaviour of a colloidal delivery system, including the pharmacokinetic profile of the loaded drugs, access to target tissues, and the overall body biodistribution, cell uptake, and clearance by Reticuloendothelial System (RES) [13].

As the batch-to-batch variability of liposome formulations yields an unpredictable fate of the carrier upon in vivo administration, approaches for scalable formulation that limit the batch variability are needed.

Formulation methods that exploit the fluid control and lipid mixing can facilitate liposome preparation in a one step process. For example, ethanol injection into an aqueous buffer [8] allows for phase separation of the lipids that spontaneously assemble into liposomes. This process, however, is usually run in a bulk reactor where the mixing of solutions is not finely controlled. More recently, microfluidic techniques where fluid mixing is performed in a geometrically constrained microenvironment, typically defined by sub-micrometre length scale and low Reynolds number, have been developed [14,15]. The rapid microfluidic mixing at the nano-litre scale allows for the nano-assembling of lipids into liposomes [16]. Indeed, the microfluidic technology efficiently controls the parameters affecting the assembly and yields highly reproducible formulations. The laminar flow of aqueous and organic solvent streams and predictable flow patterns in the microfluidic mixing result in a uniform particle size distribution. This technique allows also for controlling the size of the liposomes by setting-up proper operative conditions, namely total flow rate (TFR), flow rate ratio (FRR) of the solutions mixed, total volume (TV) processed, and solvent selection [17]. The identification of the parameters that affect the liposome features is crucial when a formulation is generated with a new procedure, namely the microfluidic, with the aim to replace an existing one.

In the present study, we investigated the effect of the formulation parameters on the biopharmaceutical properties of dexamethasone hemisuccinate loaded liposomes prepared by thin-film hydration and microfluidic techniques using a calcium acetate mediated remote loading process.

## 2. Results

### 2.1. Liposome Characterization

Dexamethasone free liposomes (plain liposomes), generated by thin-film hydration with different calcium acetate concentrations, were extruded through a 100 nm cut-off membrane to reduce the size and polydispersity. After extrusion, the liposomal diameter of the preparations was in the range of 144.1 ± 13.8–162.7 ± 17.0 nm and the poly dispersity index (PDI) was in the range of 0.08–0.39. The higher liposome size with respect to the filter pore size is due to the reversible elastic deformation of the vesicles as reported in the literature [18].

In the case of the microfluidic process, a pre-formulation study was carried out to set-up the operative conditions to generate liposomes of approximately 100 nm diameter. The size of dexamethasone free liposomes (plain liposomes) generated with different calcium acetate concentrations was in the range of 105.7 ± 12.5–118.4 ± 21.5 and PDI in the range of 0.17–0.28. Flow rate ratios (FRR) higher than of 1.5:1 aqueous/methanol, and a total flow rate (TFR) lower than 3 mL/min generated liposomes with a size above 200 nm, a high PDI, and low calcium acetate loading.

The calcium acetate concentration used for the liposome preparation did not affect the liposome size obtained by the two techniques.

### 2.2. Loading Efficiency and Capacity

**Figure 1:**
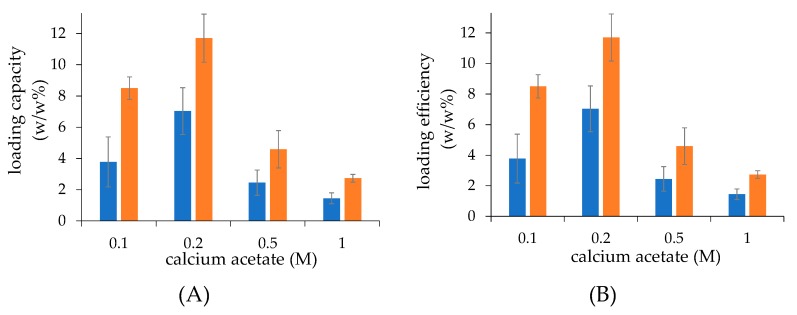
The loading capacity (**A**) and efficiency (**B**) of dexamethasone hemisuccinate in liposomes assembled at increasing concentrations of calcium acetate. The liposomes were obtained by thin-film hydration (■) and microfluidic (■) techniques. Calcium acetate loaded liposomes and dexamethasone hemisuccinate were incubated at 2.5 mg/mL, respectively. The loading capacity was expressed as loaded drug/lipid *w/w*% ratio; the loading efficiency was expressed as (loaded drug/fed drug) *w/w*% ratio.

The effect of calcium acetate concentration used to generate plain liposomes (from 0.1 to 1.0 M) on drug loading capacity and efficiency was first assessed. Both formulations obtained by thin-film hydration and microfluidics showed that the loading capacity and efficiency of dexamethasone increased as the calcium acetate concentration used to assemble liposomes increased from 0.1 M to 0.2 M, which was expected as a higher amount of calcium ions in the liposome core will entrap more dexamethasone in the liposomes during remote loading. On the contrary, at concentrations above 0.2 M calcium acetate, the loading capacity and efficiency decreased (Figure 1A,B). In this case, the loading capacity and efficiency have the same values because the study was performed using the same lipid and dexamethasone hemisuccinate concentration (2.5 mg/mL).

At all calcium acetate concentrations used for the preparation of plain liposomes, the dexamethasone loading in liposomes obtained by the microfluidic procedure was found to be higher than in the case of liposomes produced by the thin-film hydration technique.

Under the drug loading conditions, liposomes produced with 0.2 M calcium acetate by microfluidic and thin-film hydration procedures yielded the maximal 11.7 and 7.0 drug/lipid *w/w*% loading capacities, respectively, corresponding to 67% higher loading in the case of liposomes obtained by microfluidics. Based on these results, 0.2 M calcium acetate was selected for preparing liposomes for the further investigations.

**Figure 2:**
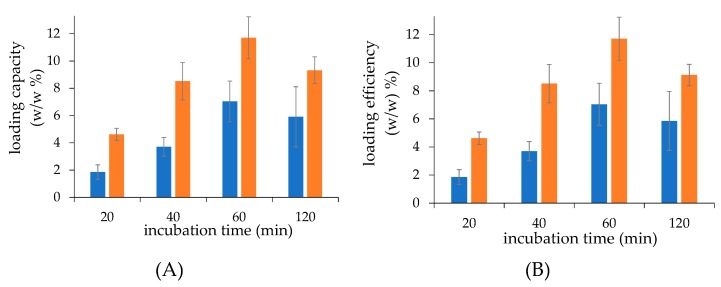
The loading capacity (**A**) and efficiency (**B**) of dexamethasone hemisuccinate in calcium acetate pre-loaded liposomes at increasing incubation times of the liposomes with the drug. Liposomes were obtained by thin-film hydration (■) and microfluidic (■) techniques. Calcium acetated loaded liposomes and dexamethasone hemisuccinate were incubated at 2.5 mg/mL, respectively. The loading capacity was expressed as loaded drug/lipid *w/w*% ratio; the loading efficiency was expressed as (loaded drug/fed drug) *w/w*% ratio.

A drug loading time course study showed that the dexamethasone loading efficiency and capacity increased with the incubation time until 60 min, either in the case of liposomes prepared by thin-film hydration or microfluidics (Figure 2), which corresponds to the maximal 11.7 and 7.0 drug/lipid *w/w*% loading capacity for the liposomes obtained by microfluidics and by thin-film hydration, respectively, as discussed for Figure 1. Once again, the microfluidic procedure allowed for a higher loading capacity and efficiency at each timepoint with respect to the thin-film hydration generated liposomes. To note that similarly to what was reported for Figure 1, the loading capacity and efficiency have the same values because the study was performed using the same lipid and dexamethasone hemisuccinate concentration (2.5 mg/mL).

**Figure 3:**
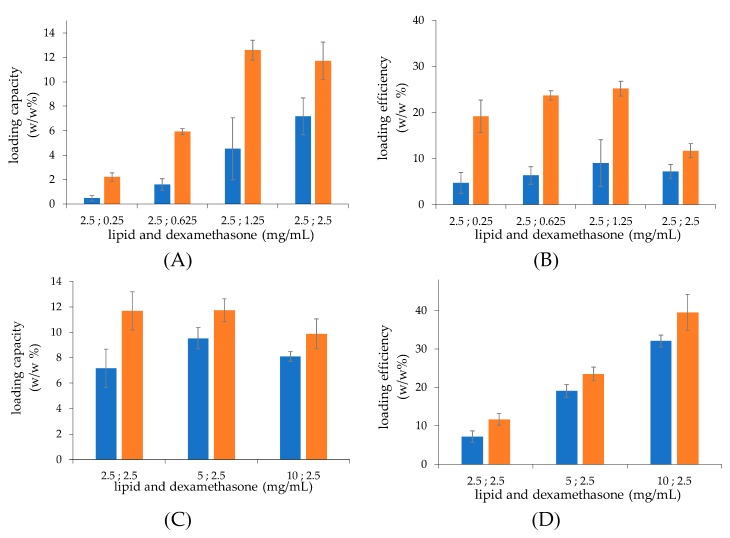
The loading capacity (**A**,**C**) and efficiency (**B**,**D**) of dexamethasone hemisuccinate in liposomes at a fixed lipid concentration of 2.5 mg/mL (**A**,**B**) and a fixed dexamethasone hemisuccinate concentration of 2.5 mg/mL (**C**,**D**) while varying their weight ratio. Liposomes were obtained by thin-film hydration (■) and microfluidic (■) techniques. The loading capacity was expressed as loaded drug/lipid *w/w*% ratio; loading efficiency was expressed as (loaded drug/fed drug) *w/w*% ratio.

The effect of the lipid/dexamethasone hemisuccinate fed ratio on the loading process was evaluated by either maintaining a constant lipid concentration and increasing the drug concentration or maintaining a constant drug concentration and increasing the lipid concentration (Figure 3). Under a constant lipid concentration, the loading capacity (Figure 3A) increases as the drug concentration increases to reach the maximal values at 1.25 mg/mL drug concentration, yielding the maximal 12.6 and 4.5 drug/lipid *w/w*% loading capacity for liposomes obtained with microfluidic and thin-film hydration, respectively, which corresponds to a 179% higher loading for the liposomes obtained by microfluidics. The drug loading efficiency (Figure 3B) agreed with the capacity profiles. Indeed, the loading efficiency increased up to the plateau and then decreased.

When the lipid concentration is increased, more vesicles are present in the medium at a constant drug concentration, which results in a high total drug loading and a constant capacity (lipid/drug ratio) is achieved (Figure 3C). The higher the number of vesicles obtained by the lipid concentration increase, the higher the amount of drug loaded in the vesicles, which results in an increased efficiency of the process (Figure 3D). Microfluidic and thin-film hydration procedures yielded a maximal 11.7 and 7.0 drug/lipid *w/w*% loading capacity, respectively, which corresponds to a 67% higher loading for the liposomes obtained by microfluidics than those generated by thin-film hydration.

Overall, this study shows that drug loading can be optimised by the selection of proper conditions. The liposomes obtained by microfluidics yielded a higher drug loading capacity with respect to the formulation obtained by thin-film hydration. Based on these results, the 10 mg/mL lipid formulations obtained with 0.2 M calcium acetate and loaded by 60 min incubation with 2.5 mg/mL dexamethasone hemisuccinate were selected to obtain the highest dexamethasone concentration: 0.81 and 0.98 mg/mL for liposomes obtained by thin-film hydration and microfluidics, respectively.

### 2.3. Size, Morphology, and Colloidal Stability of Dexamethasone Loaded Liposomes

**Figure 4:**
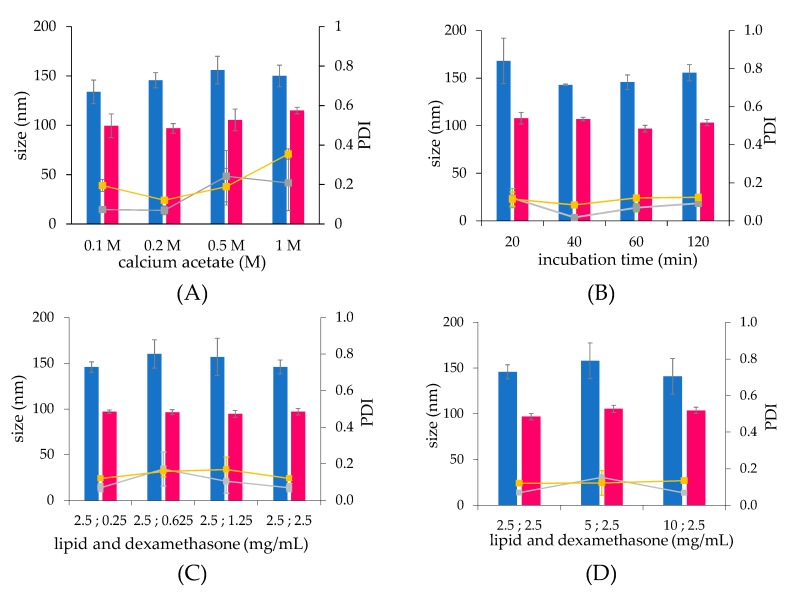
The size of dexamethasone hemisuccinate loaded liposomes obtained by thin-film hydration (■) and microfluidic (■) techniques and the corresponding PDI (■, and ■, respectively). (**A**) liposomes assembled at increasing calcium acetate concentration and loaded by contact of 2.5 mg/mL of lipids and dexamethasone hemisuccinate; (**B**) liposomes loaded at increasing contact time with dexamethasone hemisuccinate by contact of 2.5 mg/mL of lipids and dexamethasone hemisuccinate,; (**C**): liposomes loaded at increasing dexamethasone concentration and fixed lipid concentration (mg/mL); (**D**) liposomes loaded at increasing lipid concentration and fixed dexamethasone concentration (mg/mL).

The incubation of liposomes with dexamethasone hemisuccinate during the remote loading process did not induce a size increase of liposomes (Figure 4A). Furthermore, neither the incubation time, nor the lipid/drug ratio affected the vesicle size and polydispersity (Figure 4B–D). In general, the formulation obtained by thin-film hydration procedure provided larger batch-to-batch differences, which reflected in the larger standard deviation with respect to that obtained by microfluidic liposome preparation.

Formulations obtained by thin-film hydration and microfluidic procedures under the optimized conditions reported above, with size of 140.9 ± 19.5 nm (PDI 0.07) and 103.7 ± 3.5 nm (PDI 0.13) (Figure S1), respectively, were characterized.

**Figure 5:**
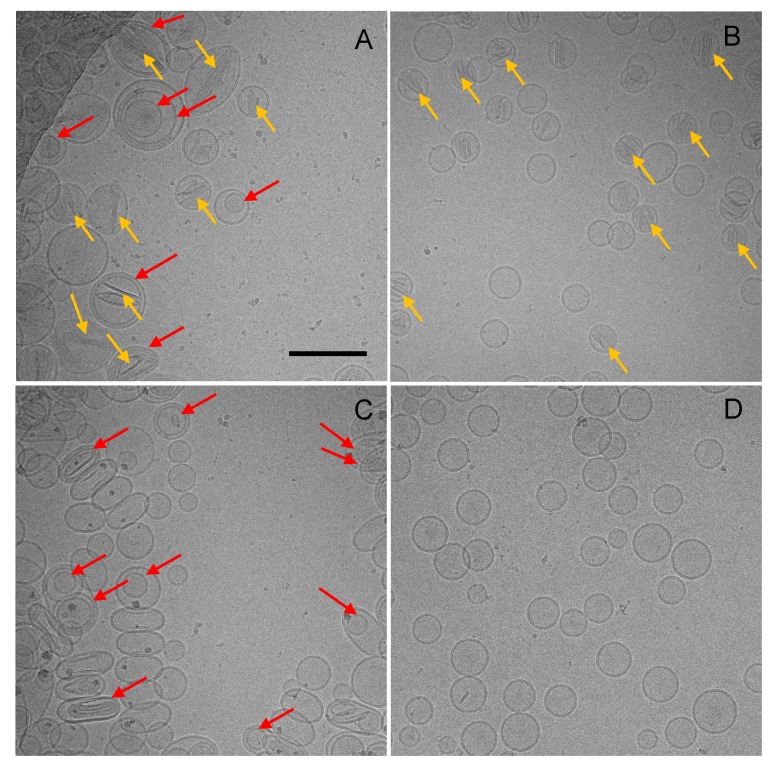
Cryogenic transmission electron microscopy (Cryo-TEM) image of liposomes obtained by thin-film hydration (**A**,**C**) and microfluidic (**B**,**D**) techniques. A and B: liposomes loaded with dexamethasone hemisuccinate. C and D: dexamethasone free liposomes (plain liposomes). Red arrows indicate lamellae in multilamellar vesicles, and yellow harrows indicate calcium-dexamethasone hemisuccinate nanocrystalline complexes. Liposomes were assembled with 0.2 M calcium acetate and loaded by incubation for one hour with dexamethasone hemisuccinate at a 10 and 2.5 mg/mL concentration of lipid and drug, respectively. Size bar: 200 nm.

In all cases, the liposome diameters observed by cryogenic transmission electron microscopy (Cryo-TEM) well correlated with the Dynamic Light Scattering (DLS) measurements. The Cryo-TEM images of liposomes obtained with the two procedures showed vesicular structures with a darker liposome membrane surrounding the inner aqueous compartment containing darker rod-shaped structures corresponding to the nanocrystalline calcium-dexamethasone hemisuccinate complex (Figure 5 and Figure S2). This was in agreement with the observations reported in the literature [19]. The liposomes obtained by thin-film hydration and hand extrusion showed a non-homogeneous multilamellar structure (Figure 5A,C) while the liposomes obtained by microfluidic procedure appeared as homogeneous unilamellar vesicles (Figure 5B,D).

**Figure 6:**
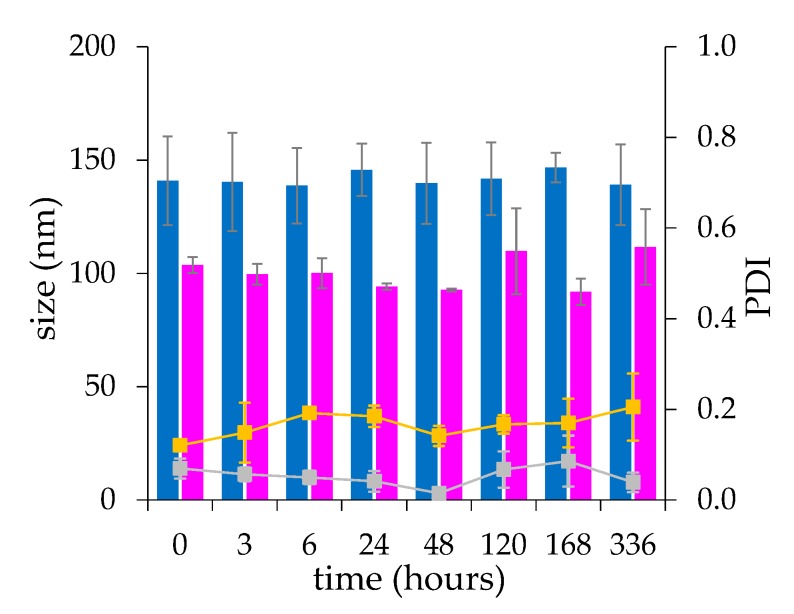
The stability profile of liposomes obtained by thin-film hydration (■) and microfluidic (■) procedures and the corresponding PDI (■ and ■, respectively). The size was measured by DLS. Liposomes were assembled with 0.2 M calcium acetate and loaded by incubation for one hour with dexamethasone at a 10 and 2.5 mg/mL concentration of lipid and drug, respectively. The data are presented as mean ± SD (*n* = 3).

The liposome formulations were fairly stable over two weeks incubation at room temperature (Figure 6).

### 2.4. In vitro Release

**Figure 7:**
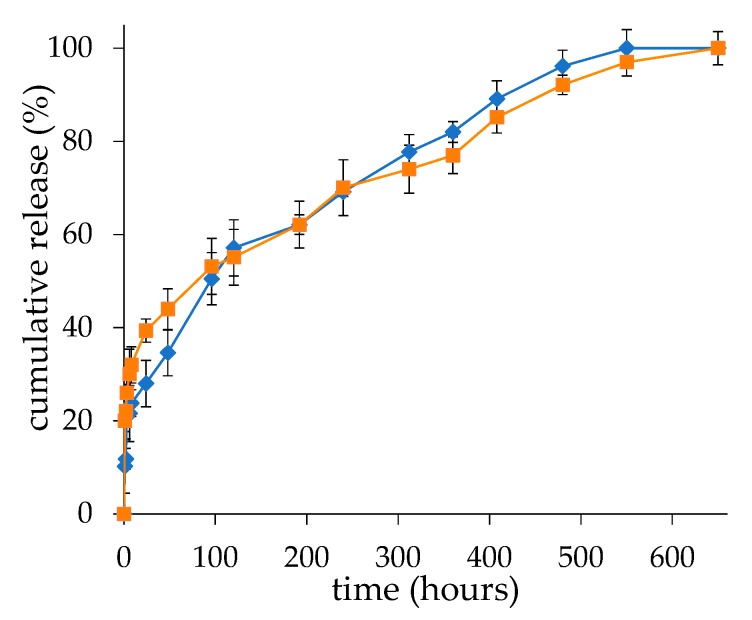
Dexamethasone release profiles of liposomes obtained by thin-film hydration (■) and microfluidic (■) techniques in 20 mM HEPES, 150 mM NaCl, pH 7.4. The data are presented as mean ± SD (*n* = 3).

The drug release studies carried out under physiological pH and osmolarity showed that the two formulations have similar release profiles with an initial burst in the first 24 h followed by a slow release (Figure 7). However, the liposomes produced by microfluidic techniques showed a higher burst than the ones produced by thin-film hydration. In both cases the remaining drug was released in about four weeks, which is consistent with the slow dissolution of the nanocrystalline calcium-dexamethasone hemisuccinate complexes into the vesicles.

### 2.5. In vitro Biological Studies

The in vitro cytotoxicity of dexamethasone loaded liposomes was studied on ARPE-19 retina cells, which represents a consolidated cell model for dexamethasone activity assay. The retinal pigment epithelium (RPE) undergoes oxidative stress, which induces local inflammation in the eye correlated to Age-related Macular Degeneration (AMD). This is responsible of severe vision loss in aged individuals in developed countries [20]. Thus, the exploitation of novel delivery systems, such as liposomes, for the local administration of anti-inflammatory corticosteroids can be beneficial for the improvement of existing treatments and patient compliance.

**Figure 8:**
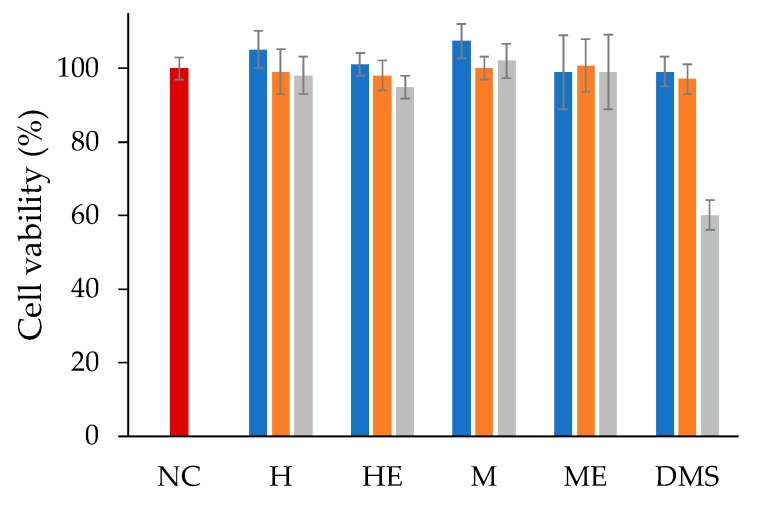
The in vitro cytotoxicity profile of dexamethasone hemisuccinate loaded liposomes obtained by thin-film hydration and microfluidic procedures. The concentration refers to the dexamethasone hemisuccinate or equimolar lipid concentration in the case of empty liposomes with respect to drug loaded liposomes: 1 µM (■), 10 µM (■), and 100 µM (■). Sample identity: NC, negative control-non treated cells; H, dexamethasone hemisuccinate loaded liposomes obtained by thin-film hydration; HE, dexamethasone free liposomes obtained by thin-film hydration; M, dexamethasone hemisuccinate loaded liposomes obtained by microfluidics; ME, dexamethasone free liposomes obtained by microfluidics; DMS, dexamethasone hemisuccinate in solution. The data are presented as mean ± SD (*n* = 3).

The cell culture studies showed that both dexamethasone free liposomes and dexamethasone loaded liposomes obtained by thin-film hydration and microfluidics were not cytotoxic. Indeed, 24 h of cell incubation, which corresponds to the burst release time, did not elicit cytotoxicity even at a high drug concentration (Figure 8). According to the literature, in the case of control cells incubated with an equivalent concentration of free dexamethasone hemisuccinate, cytotoxicity was observed only at a high drug concentration (100 µM) [21,22].

**Figure 9:**
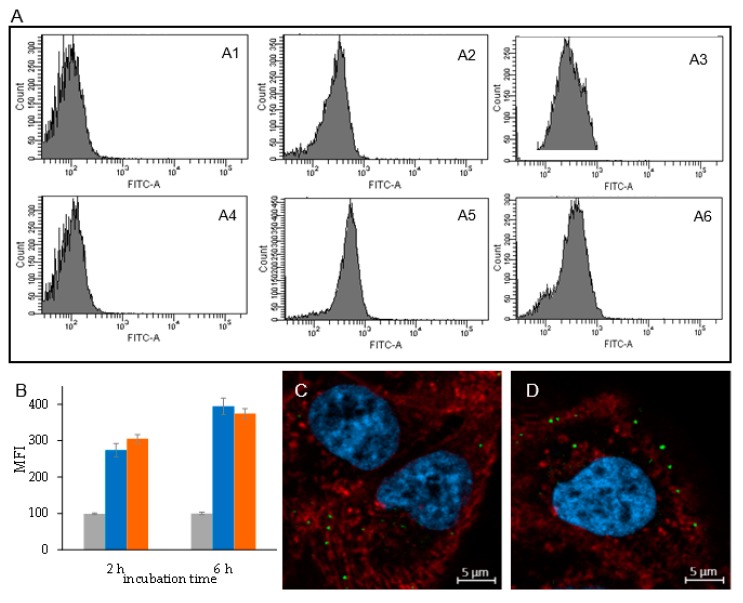
(**A**) The cytofluorimetric profiles of ARPE-19 cells incubated with fluorescently labelled dexamethasone hemisuccinate loaded liposomes obtained by thin-film hydration (A2 and A3) and by microfluidic techniques (A5 and A6) incubated for 2 (A2 and A5) and 6 (A3 and A6) hours. Untreated cells were incubated for 2 (A1) and 6 (A4) hours with culture medium. (**B**) Mean Fluorescence Intensity (MFI) of ARPE-19 cells incubated with fluorescently labelled dexamethasone hemisuccinate loaded liposomes obtained by thin-film hydration (■) and microfluidic (■) techniques and control untreated cells (■) (*n* = 3). Confocal images of ARPE-19 cells incubated with dexamethasone hemisuccinate loaded liposomes obtained by thin-film hydration (**C**) and microfluidics (**D**); liposomes were labelled with fluorescein-DHPE (green), cell membrane with wheat germ agglutinin-Alexa fluor-633 (red), nuclei with DAPI (blue). Bright field is reported in Figure S3.

The cytofluorimetric study showed that the liposomes obtained either by thin-film hydration or by microfluidic techniques undergo time dependent association to ARPE-19 cells (Figure 9A,B), which is in agreement with the literature [23,24]. No significant difference in the association of the vesicles to the cells was detected for the two formulations, indicating that the size and structural differences of the two products do not affect the interaction with cells. Confocal microscopy showed that the fluorescein labelled liposomes associate to the cells and are mostly detected as green fluorescent spots either in the proximity of the cell membrane or in the cytosol (Figure 9C,D). The similar density in cells treated with liposomes obtained by thin-layer hydration and microfluidic techniques agrees with the results obtained by the cytofluorimetric analysis.

**Figure 10:**
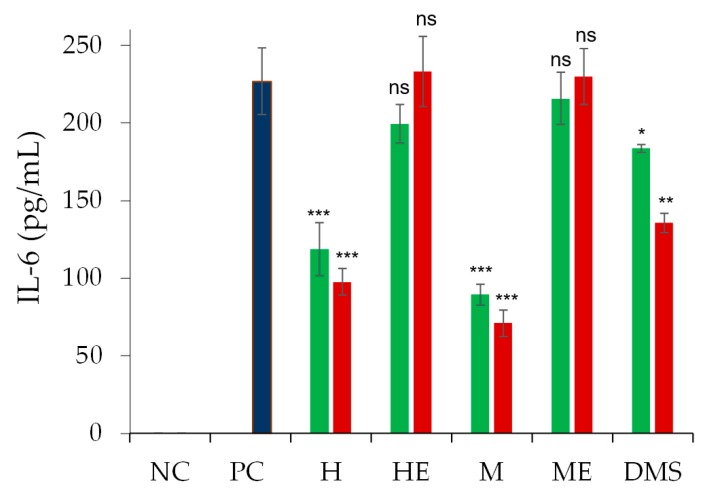
The in vitro IL-6 release by ARPE-19 cells after stimulation with lipopolysaccharide (LPS) and treatment with liposomes. Cells were treated with dexamethasone hemisuccinate loaded liposomes at 1 µM (■) and 10 µM (■) drug concentrations or an equimolar concentration of empty liposomes. Abbreviations: NC, negative control (non-treated cells); PC, positive control (cells treated only with LPS); H, pre-stimulated cells treated with dexamethasone hemisuccinate loaded liposomes obtained by thin-film hydration; HE, pre-stimulated cells treated with empty liposomes obtained by thin-film hydration; M, pre-stimulated cells treated with dexamethasone hemisuccinate loaded liposomes obtained by microfluidics; ME, pre-stimulated cells treated with empty liposomes obtained by microfluidics; and DMS, pre-stimulated cells treated with free dexamethasone hemisuccinate in solution. The data are presented as mean ± SD (*n* = 3). *P* > 0.0.5, ns; *P* ≤ 0.05, *; *P* ≤ 0.01, **; *P* ≤ 0.001, *** with respect to the positive control (PC). Additional statistical analysis was performed on dexamethasone loaded liposomes vs. free dexamethasone hemisuccinate and plain liposomes (drug free) obtained by thin-film hydration vs. plain liposomes obtained by microfluidics (See Table S1 in Supplementary Materials).

The anti-inflammatory activity of the formulations was assessed by assaying the inhibition of pro-inflammatory cytokine, namely Interleukin 6 (IL-6) released by lipopolysaccharide (LPS) stimulation of ARPE-19 cell, induced by 0, 1, and 10 μM dexamethasone loaded liposomes [25]. Empty liposomes produced with the two techniques did not present anti-inflammatory activity (Figure 10) while the dexamethasone-loaded liposomes obtained by thin-film hydration and microfluidic techniques significantly reduced the pro-inflammatory activity induced by LPS. At 1 μM dexamethasone concentration, the liposomes obtained by thin-film hydration procedure and the liposomes obtained by microfluidics were 2.3-times and three-times more efficient than free dexamethasone, respectively. Control plain liposomes (drug free) did not display pro-inflammatory activity on non-stimulated cells (no LPS treatment; Figure S4).

## 3. Discussion

In this comparative study, two liposome formulations were produced by microfluidics and by conventional thin-film hydration. The plain liposomes were produced using calcium acetate, as calcium ions in the aqueous core of liposomes establish a Ca^2+^ gradient that provides the entrapment of dexamethasone hemisuccinate, the drug model used in this study, in the vesicle [19,26,27]. Although only certain drugs can be loaded in liposomes by remote loading, the opportunity to encapsulate drugs in the liposome core by complexation with ions represents the most efficient procedure to ensure high payloads. The first drug to be encapsulated by this procedure was Doxorubicin, whose encapsulation in liposomes was patented back in the 1990s [28]. Throughout the years, other drugs have been successfully encapsulated by remote loading, including corticosteroids [27]. According to the physicochemical features, amphipathicity, and low acidic character, dexamethasone hemisuccinate is an excellent candidate for remote loading into liposomes [29].

The light scattering and Cryo-TEM analysis showed that the thin-film hydration procedure yields larger morphologically heterogeneous multilamellar liposomes, while smaller homogeneous unilamellar vesicles are obtained with the microfluidic procedure. This proves that the microfluidic approach allows for a more structure-controlled formulation. Furthermore, at the same lipid concentration, multilamellar liposomes possess a higher amount of lipid molecules per liposome vesicle with respect to unilamellar liposomes, which account for a smaller aqueous volume normalized for the lipids and, in turn, a lower drug loading capacity (expressed as drug/lipid *w/w*% ratio).

The drug loading was found to be affected by the calcium concentrations used for the liposome preparation. Liposomes obtained by the two preparation procedures showed that the drug loading capacity and efficiency increased as the calcium concentration increased to reach the maximal values at 0.2 M, and then decreased at higher calcium concentrations. This behaviour could be attributed to the hyperosmotic condition of the liposome core when liposomes are dialysed vs. isosmotic buffer to remove non encapsulated calcium acetate, which may alter the membrane permeability and cause partial loss of the calcium acetate of the liposome aqueous core.

The time course of drug loading profiles were in agreement with the literature, which reports that the drug loading by remote control occurs in 1 h [19] while a longer incubation may decrease the loading because of slow dissipation of the ion gradient from the liposome core [30].

The incubation of liposomes with increasing concentrations of dexamethasone hemisuccinate yielded a plateau in drug loading capacity, which is achieved with liposomes prepared by using 1.25 mg/mL dexamethasone-hemisuccinate. As a consequence, in the case of liposomes prepared with a higher drug concentration, the drug loading efficiency dramatically decreases. The maximal drug loading capacity with 1.25 mg/mL dexamethasone hemisuccinate may be attributable to the intravesicle calcium consumption by drug complexation. On the other side, when the lipid concentration increases, the loading capacity expressed as loaded drug/lipid *w/w*% ratio does not increase. Indeed, the increase of lipid concentration results in the increase of concentration of liposome particles but not in the increase of the liposome drug payload (expressed as drug/lipid *w/w*% ratio).

In all preparations with the same lipid concentration, the loading capacity is higher in the case of the liposomes obtained by microfluidics with respect to those obtained by thin-film hydration, which is due to the overall higher intravesical volume of the former monolayer vesicles with respect to the multilayer vesicles produced by thin-film hydration.

The dexamethasone entrapment into the liposome core has been observed by Cryo-TEM imaging, which showed dark nanocrystals within the liposome aqueous compartment resulting in “coffee bean” like vesicles, which are similar to the vesicles obtained by remote loading of anionic corticosteroids, as reported in the literature [31]. The structures in the vesicle aqueous core indicate that the calcium dexamethasone hemisuccinate is in a “precipitated form”.

The microfluidic procedure was found to produce liposomes with low batch-to-batch variability and higher stability during the drug loading process, resulting in a higher production reproducibility compared to the liposomes produced by thin-film hydration. This evidence confirms that the microfluidic based formulation of liposomes, by virtue of the chaotic advection mixing process into the channels of a staggered herringbone micromixer (SHM) at low shear stress provides a quick and locally controlled process of assembly of the lipids [32].

The in vitro release profiles reflected the structural properties of the liposomes. The burst release of the drug in the first 24 h is attributable to the dexamethasone in solution in the liposome core, which is in equilibrium with the “nanocrystalline form” observed by Cryo-TEM. The higher burst obtained with the liposomes produced by microfluidics with respect to the ones produced by thin-film hydration is likely due to the higher dexamethasone concentration in the liposome aqueous core. Notably, for both the liposome formulations obtained with the two techniques, while the initial fast drug release is dictated by the diffusion of a soluble drug fraction from the aqueous compartment of the vesicles, the second phase of slower release is guided by the dissolution rate of the drug from the nanocrystalline structures in the aqueous core of the liposomes.

In vitro cell studies were carried out using retinal pigmented epithelium cells (ARPE-19) to obtain pharmacological information of the two liposomal formulations. The ARPE-19 cell line was selected because dexamethasone has been used to treat inflammation in a variety of ocular diseases including diabetic macular oedema, retinal vein occlusion [33,34], and in combination with anti-Vascular Endothelial Growth Factor (anti-VEGF) agents for the therapy of age related macular degeneration (AMD) [35]. Both formulations were found to be biocompatible under the experimental conditions, even at high drug concentrations, which agreed with the slow release of a major fraction of loaded drug. On the other hand, both formulations showed a high anti-inflammatory performance. The non-direct correlation of the inhibition of IL-6 release by cells with respect to the dexamethasone concentration in the medium is in agreement with the observations reported in the literature [25]. The higher activity of the liposomal formulations with respect to the free drug, despite the partial drug availability of the dexamethasone in the liposomes and the complete availability of free dexamethasone, can be attributed to the liposome capacity to associate to cells as observed by cytofluorimetry and confocal microscopy and intracellularly deliver the anti-inflammatory drug. The better performance of the liposomes obtained by microfluidics with respect to the ones obtained by thin-film hydration can be ascribed to a higher burst release in the first few hours, which can provide a higher intracellular concentration of dexamethasone.

## 4. Materials and Methods

### 4.1. Materials

Hydrogenated phosphatidyl choline from soyabeans (HSPC, ≥98%, Tm 52.5 °C) was received as a gift from Lipoid GmBH (Ludwigshafen, Germany). Cholesterol was purchased from Sigma-Aldrich (St Louis, MO, USA). The glucocorticoid pro-drug dexamethasone hemisuccinate (1,4-Pregnadien-9α-Fluoro-16α-Methyl-11β,17,21-Triol-3,20-Dione 21-Hemisuccinate) was purchased from Steraloids Inc (Newport, RI, USA). Dulbecco’s Modified Eagle’s Medium/nutrient mixture F-12 without glutamine was purchased from Aurogene (Rome, Italy). Penicillin-Streptomycin solution (10,000 units penicillin and 10 mg streptomycin/mL), L-glutamin (200 mM), Trypsin (10×), Lipopolysaccharides from *Escherichia coli* O111:B4 (LPS), 3-(4,5-Dimethylthiazol-2-yl)-2,5-diphenyltetrazolium bromide (MTT), Fetal Bovine Serum (FBS), and Phosphate Buffered Saline (PBS) were purchased from Sigma (St Louis, MO, USA). N-(fluorescein-5-thiocarbamoyl)-1,2-dihexadecanoyl-sn-glycero-3-phosphoethanolamine, triethylammonium salt (Fluorescein-DHPE) was purchased from Biotium Inc. (Fremont, CA, USA). Wheat germ agglutinin (WGA) Alexa Fluor 633 conjugate was purchased from Molecualr Probes (Eugene, OR, USA). 4′,6-Diamidino-2-phenylindole (DAPI) was purchased from Vector laboratories, Inc. (Burlingame, CA, USA). An IL-6 ELISA duo set kit was purchased from R&D systems (Minneapolis, MN, USA). The water used for all experiments was produced with the Millipore Milli-Q purification system (Massachusetts, MA, USA) and filtered sterilized. All chemicals used in this study were of high analytical grade.

### 4.2. Assembly of Liposomes

Dexamethasone hemisuccinate loaded liposomes were formulated with a two-step process: In the first step, calcium acetate loaded liposomes were assembled and then they were incubated with dexamethasone hemisuccinate for remote loading.

Calcium acetate loaded liposomes were either fabricated by conventional thin-film hydration or by a microfluidic process.

Thin-film hydration process. Liposomes were prepared according to the thin-film hydration process reported in the literature [1]. Briefly, a stock solution of HSPC and cholesterol in CHCl_3_ was prepared at a concentration of 6.67 mg/mL. A 2:1 HSPC/Cholesterol molar ratio solution was generated in a 10 mL round bottom flask by mixing two stock solutions (2405.2 µL of HSPC and 593.3 µL cholesterol resulting in 20 mg lipid). The organic solvent was removed under reduced pressure at 37 °C using a rotary evaporator. Then the lipid film was hydrated with 1 mL of calcium acetate solution in MQ water at different concentrations (a 0.1–1 M range). The lipidic vesicle suspension underwent 10 freeze-and-thaw cycles and extrusion after each cycle through a polycarbonate membrane with pores of 100 nm cut-off diameter using an Avanti mini extruder (Avanti Polar Lipids Inc. Alabaster, AL, USA).

Microfluidic process. Liposomes were also prepared using a NanoAssemblr^®^ Benchtop system (Precision Nano System, Vancouver, BC, Canada). Briefly, a 2:1 HSPC/Cholesterol molar ratio mixture in methanol was generated at a concentration of 30 mg/mL. Calcium acetate solution in milliQ water in the concentration range of 0.1–1 M was used as aqueous phase. Liposomes were assembled by processing 1 mL of the lipidic organic solution and 1.5 mL of the calcium acetate solution using a benchtop NanoAssembler system equipped with a Staggered Herringbone Mixing cartridge resulting into liposome assembly by nano-precipitation [36]. The Total volume (TV), Flow rate ratio (FRR) between the aqueous and organic stream, and total flow rate (TFR) were set to 2.5 mL, 17 mL/min, and 1.5:1, respectively, to generate calcium acetate liposomes.

Methanol was removed from the formulations by rotary evaporation under reduced pressure for 6 min at 35 °C. The complete removal of methanol was confirmed by ^1^H NMR analysis with a Bruker 400 MHz NMR Avance spectrometer (Billerica, MA, USA).

### 4.3. Remote Loading of Dexamethasone Hemisuccinate

We dialyzed 1 mL of freshly prepared calcium acetate loaded liposomes formulated by thin-film hydration or microfluidic procedures (20 mg/mL lipids) against 1 L of 20 mM HEPES, 150 mM NaCl, pH 7.4 overnight using a 100 Kda float-a-lyzer to remove non loaded calcium acetate and generate a calcium gradient. The resulting lipid concentration after dialysis was assessed by the Stewart assay [37]. Liposomes were diluted with the same buffer when needed.

A 5 mg/mL dexamethasone hemisuccinate stock solution was prepared in 20 mM HEPES, 150 mM NaCl, pH 7.4.

Incubation time effect on loading. A dedicated study was performed to elucidate the effect of the incubation time on remote loading. We pre-incubated 500 µL volume of 5 mg/mL dexamethasone hemisuccinate solution and 500 µL samples of 5 mg/mL calcium acetate loaded liposomes (obtained using 0.2 M calcium acetate) at 65 °C for 5 min and then mixed them together. The resulting liposomes/dexamethasone hemisuccinate mixture was incubated for 20, 40, 60, or 120 min at 65 °C and then cooled down at 4 °C. Non-loaded dexamethasone hemisuccincate was removed by dialysis against 1 L of 20 mM HEPES, 150 mM NaCl, pH 7.4 overnight using a 100 KDa float-a-lyzer.

Lipid/dexamethasone hemisuccinate ratio effect on loading. 500 µL volume of dexamethasone hemisuccinate solutions at increasing concentrations from 0.5, to 5 mg/mL and 500 µL samples of 5 mg/mL calcium acetate loaded liposomes (obtained using 0.2 M calcium acetate) were pre-incubated at 65 °C for 5 min and then each of the dexamethasone hemisuccinate solutions was added and mixed to one liposome sample and mixtures were incubated for 60 min at 65 °C and then cooled down at 4 °C.

Additionally, 500 µL of 5 mg/mL dexamethasone hemisuccinate solutions and calcium acetate loaded liposomes (obtained using 0.2 M calcium acetate) at increasing lipid concentrations from 5 to 20 mg/mL were pre-incubated at 65 °C for 5 min. Then the dexamethasone hemisuccinate solution was added and mixed to each of the liposome samples and mixtures were incubated for 60 min at 65 °C and then cooled down at 4 °C.

Non-loaded dexamethasone hemisuccincate was removed by dialysis as reported above.

The concentration of lipids after dialysis was assessed by a Stewart assay.

A validation assay of the dialysis process was performed by dialysing a 2.5 mg/mL solution of dexamethasone hemisuccinate against 1 L of 20 mM HEPES, 150 mM NaCl, pH 7.4 with the same procedure.

### 4.4. Loading Efficiency and Capacity Assessment

The dexamethasone hemisuccinate loaded into liposomes was quantified by spectrophotometric analysis. We diluted 20 μL of drug loaded liposomes with 800 µL of methanol and sonicated for 30 min in a water bath sonicator to disassemble the liposomes and dissolve the drug. Afterward, dexamethasone concentration was assessed by spectrophotometric analysis at 242 nm. All experiments were performed in triplicate.

The loading capacity and loading efficiency were calculated using the following formula:

*Loading Capacity (lipid/drug w/w) (%)* = [Dexamethasone encapsulated in liposomes (mg/mL) ÷ Lipids (mg/mL)] × 100(1)

*Loading Efficiency (w/w) (%)* = [Dexamethasone encapsulated in liposomes (mg) ÷ processed Dexamethasone (mg)] × 100(2)

### 4.5. Size Analysis and Stability

The size and poly-dispersity index (PDI) of liposomal formulations were measured by photo correlation spectroscopy (PCS) using a Zetasizer NanoZS (Malvern Instrument LTD, Malvern, UK). The liposomes were diluted before analysis with 20 mM HEPES, 150 mM NaCl, pH 7.4 to a concentration of 0.5 mg/mL. The size analysis was based on the Intensity.

The liposome colloidal stability was performed by incubating 10 mg/mL drug loaded liposomes in 20 mM HEPES, 150 mM NaCl, pH 7.4 at 25 °C for 14 days. The liposomes were diluted to 0.5 mg/mL with 20 mM HEPES, 150 mM NaCl, pH 7.4 at scheduled time points before analysis and the size and poly dispersity index (PDI) were measured as reported above.

### 4.6. Cryogenic Transmission Electron Microscopy (Cryo-TEM)

We applied 3.5 μL of dexamethasone hemisuccinate loaded liposomes and dexamethasone hemisuccinate free liposomes at a 10 mg/mL lipid concentration on copper 300-mesh Quantifoil R2/1 holey carbon grid, previously glow discharged for 30 s at 30 mA using a GloQube system (Quorum Technologies, Laughton, UK). After 60 s, the grid was plunge-frozen in liquid ethane using a Vitrobot Mk IV (Thermo Fisher Scientific, Waltham, MA, USA) operating at 4 °C and 100% Relative Humidity (RH). Images of the vitrified specimens were acquired using a Talos Arctica transmission electron microscope operating at 200 kV and equipped with a CETA 16M camera (Thermo Fisher Scientific, Waltham, MA, USA). Images with applied defocus values between −2 and −4 μm were acquired with a total exposure time of 1 s and a total accumulated dose of 16 electrons per A^2^ at nominal magnifications of 22,000×, corresponding to a pixel size of 4.7 Å/pixel at the specimen level.

### 4.7. Release Study

We transferred 1 mL of 10 mg/mL freshly prepared dexamethasone hemisuccinate loaded liposomes in 20 mM HEPES, 150 mM NaCl, pH 7.4 into a 20 KDa MWCO Spectra/Por^®^ Float-ALyzer^®^ and dialyzed against 2 L of the same buffer. The buffer was thermostated at 37 °C throughout the study. At scheduled time intervals, 10 μL of liposomes were withdrawn and after dilution in 500 µL of methanol dexamethasone, the concentration was assessed by spectrophotometric analysis. The release study was carried out for 30 days. The released percentage of dexamethasone was plotted versus time.

### 4.8. In vitro Cytotoxicity

The in vitro cytotoxicity of dexamethasone hemisuccinate loaded liposomes was performed by incubating the human retinal pigmented epithelia cell line (ARPE-19) with the formulations. ARPE-19 cells were cultured at 37 °C, in 5% CO_2_ atmosphere, using DMEM/F12 medium (Gibco, Thermo Fisher Scientific, Waltham, MA, USA) supplemented with 10% FBS, 2 mM L-Glutamine, 100 IU/mL penicillin, and 100 µg/mL streptomycin. The cytotoxicity study was performed by seeding ARPE-19 cells at 3 × 10^4^ cells/well in a 96-well plate; the cells were grown for 24 h in a humidified 5% CO_2_ atmosphere at 37 °C. Then the medium was discharged, the cells were washed two times with PBS and incubated with 200 μL of liposomes or free dexamethasone hemisuccinate in DMEM/F12 without FBS at equivalent drug concentrations of 1, 10, and 100 μM. Drug free liposomes at equivalent concentrations of lipids were used as a negative control. After 24 h, the medium was removed and the cells were washed carefully with PBS and then incubated with 200 µL/well of complete medium containing 20 µL of 5 mg/mL MTT solution in PBS for three hours at 37 °C. Afterward, the medium was removed and 200 µL/well of DMSO was added. After 30 min the plate was read spectrophotometrically with a microplate reader (Microplate autoreader, EL311SK, Biotek Inc, Winooski, VT, USA) equipped with a 570 nm light filter. The analysis was performed in triplicate and cell viability was expressed as percentage referring to non-treated cells.

### 4.9. In vitro Cell Association of Liposomes

Cytofluorimetric study. ARPE-19 cells (2 × 10^5^/well) were seeded on a 24-well plate in DMEM/F12 supplemented of 10% FBS and grown for 24 h. The medium was then removed, and the cells were washed twice with PBS. We added 500 µL/well of a 0.5 mg/mL of dexamethasone loaded liposomes labelled with 0.3 mol% of fluorescein-DHPE with respect to the lipids. The cells were incubated with liposomes for 2 and 6 h. Afterward, the liposome containing medium was removed, and the cells were washed twice with PBS and then with PBS added of 10% FBS. Cells were then detached by treatment with 200 µL/well of 0.125 mg/mL trypsin for 8 min at 37 °C and then transferred in FACS tubes containing 200 µL PBS buffer, pH 7.4, 0.5% BSA, 5 mM EDTA, 2 mM NaN_3_, 4% PFA. Cells were stored at 4 °C in the dark prior to analysis. Samples underwent cytofluorimetric analysis using a BD FACS CANTO^TM^ II (BD Biosciences, San Jose, CA, USA) and the fluorescence deriving from Fluoroscein-DHPE labelled liposomes was detected using a laser emitting at 496 nm.

Confocal microscopy. ARPE-19 cells (8 × 10^4^/well) were seeded on 13 mm^2^ cover slips introduced in a 24-well plate using DMEM/F12 with 10% FBS and incubated for 24 h. The medium was then removed, and the cells were washed twice with PBS. We added 300 µL of a 0.5 mg/mL of dexamethasone loaded liposomes labelled with 0.3 mol% of fluorescein-DHPE with respect to the lipids in FBS free medium to each well, and the cells were incubated for 6 h. Afterward, the medium was removed, and the cells were washed two times with PBS and two times with PBS added of 10% FBS. The cells were then fixed by 20 min of treatment with 200 µL/well of PBS buffer, pH 7.4, containing 4% (*w/v*) PFA.

The solution was then discharged, and the cells were washed three times with PBS. The staining of cell nuclei was performed by incubating the samples with 200 µL of a 5 µg/mL solution of DAPI in PBS for 10 min. After discharging the solution, the cell membranes were labelled by incubating the cells with 200 µL/well of a 5 µg/mL solution of Wheat germ agglutinin-Alexa fluor-633 conjugate in PBS for 10 min. The solution was then discharged, and the cells were washed three times with PBS and two times with mQ water. The coverslips were mounted on a glass slide using a mounting medium prepared with 10 *w/v*% of Mowiol® 4-88 (Sigma-Aldrich (St Louis, MO, USA) in a 1:3.8 glycerol/0.13 M Tris-HCl buffer pH 8.5 *v/v* ratio mixture. The samples were maintained at 4 °C in the dark until microscopic examination.

The cell samples were imaged using a Zeiss LSM 800 confocal microscope (Carl Zeiss, Jena, Germany) equipped with a 63×, n.a. 1.4, oil immersion objective and a ZEN 2.1, blue edition software (Carl Zeiss, Jena, Germany). Lasers with emission wavelengths at 405, 488, and 640 nm were used to detect DAPI, fluorescein-DHPE, and WGA-Alexa Fluor-633, respectively. To avoid emission crosstalk, each emission fluorescence was recorded independently with a specific detector and optical cut-off filter over the entire emission spectrum of related chromophores. Image analyses were performed using ImageJ 1.47v (National Institutes of Health software package by Wayne Rasband; Bethesda, MD, USA).

### 4.10. In vitro Anti-inflammatory

The anti-inflammatory activity of dexamethasone hemisuccinate loaded liposomes was investigated by assessing the inhibition of IL-6 release by LPS stimulated ARPE-19 cells. Cells were seeded at a 1.2 × 10^5^ cells/well density in a 24-well plate with complete medium and grown for 24 h in a humidified 5% CO_2_ atmosphere at 37 °C. Then, the medium was discharged, the cells were washed two times with PBS and incubated with 500 μL/well of 5 μg/mL LPS in DMEM/F12 medium without FBS for 12 h. The LPS containing medium was then removed and the cells were washed with PBS and 500 μL/well of dexamethasone hemisuccinate loaded liposomes or free drug at 1 and 10 μM in DMEM/F12 medium without FBS was added. Drug free liposomes at equivalent concentrations of lipids were then used as a control. The cells were incubated with the samples for 24 h. The medium of each well was collected, centrifuged at 13,000 rpm for 8 min, and analysed with human IL-6 ELISA Duo Set kit (R&D systems, Inc, Minneapolis, MN, USA) according to the procedure reported by the provider to quantify the concentration of IL-6 released by the cells upon suitable dilutions.

In order to assess the potential of the carrier to induce inflammation, non-stimulated cells (no LPS treatment) were incubated with plain liposomes (no drug loaded) at lipid concentrations equivalent to those of the 1 and 10 μM dexamethasone hemisuccinate loaded liposomes for 12 h. Afterward, the liposomes containing medium was removed, the cells were washed with PBS and further incubated with medium without FBS for 24 h. The cell supernatants were then tested as reported above.

## 5. Conclusions

The comparative study reported here attempted to fill, at least in part, the lack of information regarding the exploitation of microfluidics in liposome production in comparison to traditional techniques, in view of pharmaceutical scaling developments. This study shows that the two procedures investigated for liposome preparation, namely thin-film hydration and microfluidics, yield liposomes with different colloidal features. The drug loading and release profile of the vesicles are affected by the different lamellarity, size, and internal aqueous volume of the liposomes obtained with the two processes rather than by the drug loading conditions. With respect to the traditional techniques, microfluidics offer an opportunity to significantly improve the quality of the pharmaceutical product by providing reproducible liposome production, including reduction of the post-production processing steps (i.e., sonication, extrusion, freezing, and thawing) to generate liposomes with desirable characteristics of size and homogeneity, which conceivably results in time savings. The industrial production of pharmaceutical grade products can exploit microfluidic equipment operating under Good Manufacturing Practice (GMP) conditions and can be designed to translate small batch to high-throughput productions [38]. However, comparative and extensive cost analyses for industrial production generating nanopharmaceutics for clinical applications are not yet available.

## Supplementary Materials

Supplementary Materials can be found at https://www.mdpi.com/1422-0067/21/5/1611/s1.

## Abbreviations

TFR	Total Flow rate
TV	Total Volume
FRR	Flow Rate Ratio
PDI	Poly Dispersity Index
Cryo-TEM	Cryogenic Transmission Electron Microscopy
AMD	Age Related Macular Degeneration
RPE	Retinal Pigmented Epithelium
LPS	Lipopolysaccharide
IL-6	Interleukin-6
ELISA	Enzyme Linked Immune Sorbent Assay
EPR	Enhanced Permeability and Retention
RES	Reticuloendothelial System
*E. coli*	*Escherichia Coli*
MWCO	Molecular Weight Cut-off
HSPC	Hydrogenated Soybean Phosphatidyl Choline
DMEM/F12	Dulbecco’s Modified Eagle’s Medium F12
FBS	Fetal Bovine Serum
MTT	3-(4,5-dimethylthiazol-2-yl)-2,5-diphenyl tetrazolium bromide
PBS	Phosphate Buffer Saline
HEPES	(4-(2-hydroxyethyl)-1-piperazineethanesulfonic acid
DMSO	Dimethyl Sulfoxide
PFA	Paraformaldehyde
