# Supplementary material for: Dexamethasone Loaded Liposomes by Thin-Film Hydration and Microfluidic Procedures: Formulation Challenges
Source: Int J Mol Sci. 2020 Feb 26;21(5):1611. doi: 10.3390/ijms21051611 (PMC7084920; doi:10.3390/ijms21051611)
Supplement: Supplementary file 1 [file ijms-21-01611-s001.pdf]

Supplementary Materials:

Dexamethasone Loaded Liposomes by Thin-Film Hydration and Microfluidic Procedures: Formulation Challenges

MD Al-Amin, Federica Bellato, Francesca Mastrotto, Mariangela Garofalo, Alessio Malfanti, Stefano Salmaso * and Paolo Caliceti

Department of Pharmaceutical and Pharmacological Sciences, University of Padova, Via F. Marzolo 5, 35131 Padova, Italy; md.alamin@studenti.unipd.it (M.A.-A.); federica.bellato@phd.unipd.it (F.B.); francesca.mastrotto@unipd.it (F.M.); marinagela.garofalo@unipd.it (M.G.); alessio.malfanti89@gmail.com (A.M.); paolo.caliceti@unipd.it (P.C.)
* Correspondence: stefano.salmaso@unipd.it; Tel.: +39-04-9827-1602

Table S1. Statistical analysis of IL-6 release by ARPE-19 cells activated by LPS and incubated with liposomes or free dexamethasone hemisuccinate as reported in Figure 10.

Formulations	H (1 µM)	H (10 µM)	HE (1 µM)	HE (10 µM)	M (1 µM)	M (10 µM)	ME (1 µM)	ME (10 µM)	DMS (1 µM)	DMS (10 µM)
**PC**	0.0018	0.0004	0.1738	0.96	0.0004	0.0002	0.3	0.8	0.014	0.0015
**DMS (1 mM)**	0.0028				<0.0001					
**DMS (10 mM)**	0.0023				0.0002	0.02				
**H (1 mM)**					0.05					
**H (10 mM)**						0.028				

Abbreviations: PC, Positive control (cells treated with only LPS); H, dexamethasone hemisuccinate loaded liposomes obtained by thin layer rehydration; HE, empty liposomes obtained by thin layer rehydration; M, dexamethasone hemisuccinate loaded liposomes obtained by microfluidics; ME, empty liposomes obtained by microfluidics; DMS, dexamethasone hemisuccinate in solution. Drug concentration is reported in brackets.

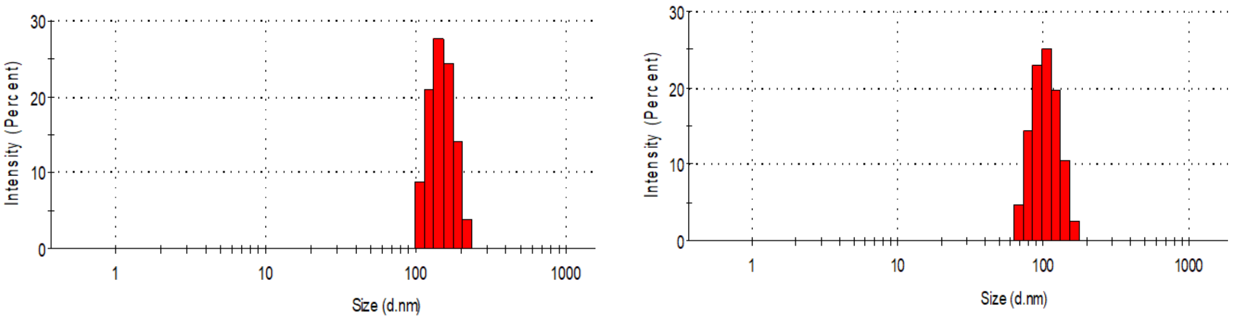

A B

Figure S1. Size distribution (by intensity) of dexamethasone-hemisuccinate loaded liposomes obtained by thin layer rehydration (A) and microfluidic (B) procedures. Liposomes were assembled with 0.2 M calcium acetate and loaded by incubation for one hour with dexamethasone hemisuccinate at 10 and 2.5 mg/mL concentration of lipids and drug, respectively.

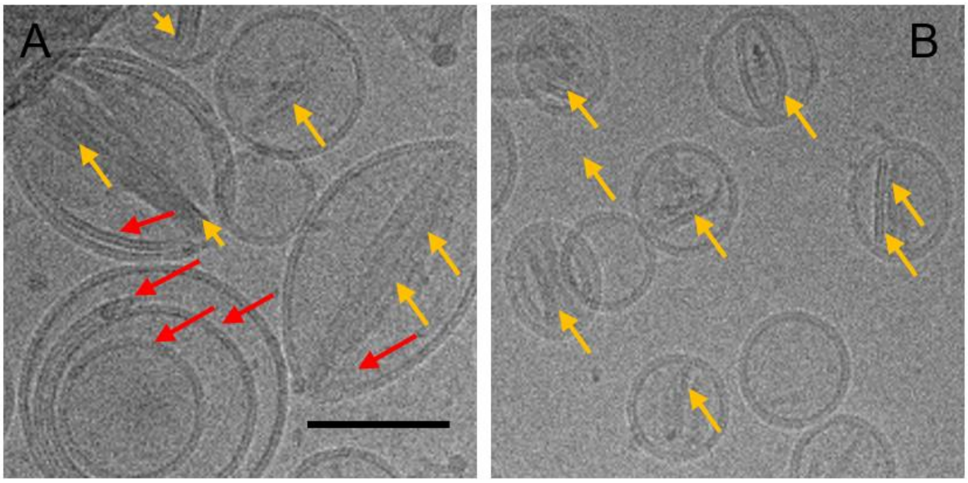

Figure S2. Cryo-TEM image of dexamethasone hemisuccinate loaded liposomes obtained by thin layer rehydration (**A**) and microfluidic (**B**). Images report a magnified field of Figure 5. Red harrows indicate lamellae in multilamellar vesicles; yellow harrows indicate calcium-dexamethasone hemisuccinate nanocrystalline complexes. Liposomes were assembled with 0.2 M Calcium acetate and loaded by incubation for one hour with dexamethasone hemisuccinate at 10 and 2.5 mg/mL concentration of lipid and drug, respectively. Size bar: 100 nm.

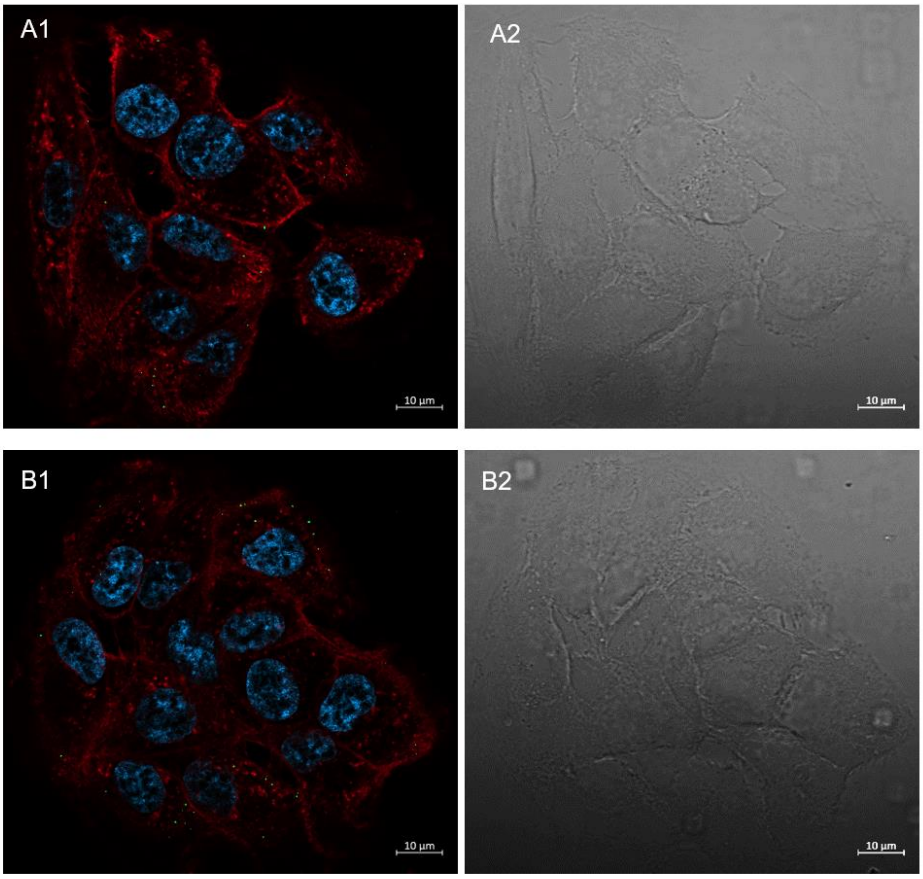

Figure S3. Confocal images of ARPE-19 cells incubated with dexamethasone hemisuccinate loaded liposomes obtained by thin-film hydration (**A1**) and microfluidics (**B1**). Liposomes were labelled with fluorescein-DHPE (green), cell membrane with wheat germ agglutinin-Alexa fluor-633 (red), nuclei with DAPI (blue). Panel **A2** and **B2** are the corresponding bright fields of images of panel A1 and B1, respectively. Images were captured on a large field from which the magnifications of Figure 9C and D were generated.

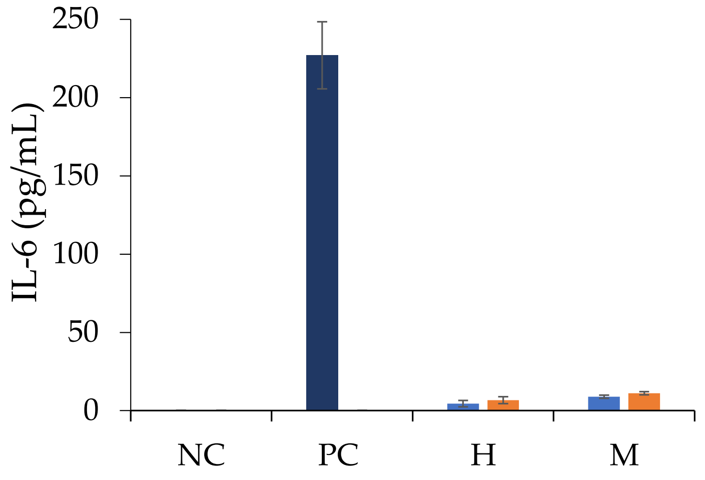

Figure S4. In vitro IL-6 release by ARPE-19 cells. Cells were treated with plain liposomes (no drug loaded) at lipid concentrations equivalent to those of the 1 (■) and 10 (■) µM Dexamethasone hemisuccinate loaded liposomes. LPS stimulation was applied only in positive control (PC). Abbreviations: NC: negative control (non-treated cells); PC: positive control (cells treated with only LPS); H: cells incubated with plain liposomes obtained by thin-layer hydration; M: cells incubated with plain liposomes obtained by microfluidics.
